# Maternally-transmitted microbiota affects odor emission and preference in Drosophila larva

**DOI:** 10.1038/s41598-017-04922-z

**Published:** 2017-07-20

**Authors:** Jean-Pierre Farine, Wafa Habbachi, Jérôme Cortot, Suzy Roche, Jean-François Ferveur

**Affiliations:** 1 Centre des Sciences du Goût et de l’Alimentation, UMR6265 CNRS, UMR1324 INRA, Université de Bourgogne Franche-Comté, 6, Bd Gabriel, 21000 Dijon, France; 2 Université Badji Mokhtar, Département de Biologie, Faculté des Sciences, 23000 Annaba, Algeria

## Abstract

Experimental studies show that early sensory experience often affects subsequent sensory preference, suggesting that the heterogeneity of sensory cues in nature could induce significant inter-individual behavioral variation, potentially contributing to maintain intraspecific diversity. To test this hypothesis, we explored the behavioral effect induced by variation in the levels of a self-produced chemical, acetoin, and its link with intraspecific diversity. Acetoin is a pheromone-like substance produced by gut-associated microorganisms in *Drosophila*. Using wild-type *Drosophila melanogaster* populations producing variable acetoin levels, we (*i*) characterized factors involved in this variation and (*ii*) manipulated some of these factors to affect acetoin responses in larvae. We found that increased and decreased variations in acetoin levels were caused by microorganisms associated with the outside and inside of the egg, respectively. Wild-type larvae preferred acetoin-rich food only when they both produced and were exposed to substantial amounts of acetoin. The removal of the outside of the egg or the genetic alteration of olfaction abolished this preference. In contrast, larvae exposed to high doses of synthetic acetoin were repulsed by acetoin. The similar effects obtained with freshly caught wild-type lines suggest that this acetoin “production-preference” link underlies the diversity of acetoin-producing microorganisms among natural *D. melanogaster* populations.

## Introduction

Acetoin (hydroxy-3-butanone-2; H_3_B_2_), a very common molecular product of the aerobic and anaerobic fermentation processes induced by microorganisms, attracts various adult insects including Drosophila^1–4^. Such attraction promotes aggregation, mating and egg-laying behaviors allowing further progeny development on the same food source^5, 6^. Some of the microorganisms involved in fermentation processes are ingested by larvae and adults feeding on such microbiota (=yeast + bacteria + mold). Yeast and bacteria, acquired from the external environment by first-instar Drosophila larvae, can persist through metamorphosis in adults^7–9^. During mating, male and female flies exchange microbiota that are subsequently deposited on breeding sites with eggs and feces^7, 10–12^. Beside diet, which can affect microbiota composition^13^, the density of bacteria species varies during development, both on the external surface and in the gut of larvae and adults^7, 14–17^.

The host-microbiota relationship is complex: mutually beneficial interactions between species play a pivotal role in most ecosystems and are only maintained when the fitness of both partners is enhanced. For example, Drosophila can manipulate the microbial community developing in fruits to increase its fitness^3, 18, 19^. Yeast-derived volatiles also attract insects^3, 20–23^ and their dynamic coexistence with varied bacteria species enhances the attractive effect of volatile compounds^24, 25^.

Our study aims to unravel some of the mechanisms involved in the relationship between the production of acetoin and the behavioral response induced by this compound. Given that most Drosophila studies pertaining to acetoin production and response were performed on adults, we focused our study on larvae. In particular, we measured the variation for the production and behavioral response to acetoin in freshly caught and lab-established *D. melanogaster* strains. Based on the extreme quantitative variation of acetoin found between and within strains, we investigated and manipulated the factors potentially underlying this variation and measured the consequence of such variation/manipulation on larval behavior.

## Results

Since our main goal aimed to discover a link between acetoin production and preference, we (*i*) investigated and manipulated some of the factors underlying the variation for acetoin production and (*ii*) measured the behavioral response to acetoin in larvae exposed to different acetoin levels.

### Natural variation for acetoin production

**Figure 1:**
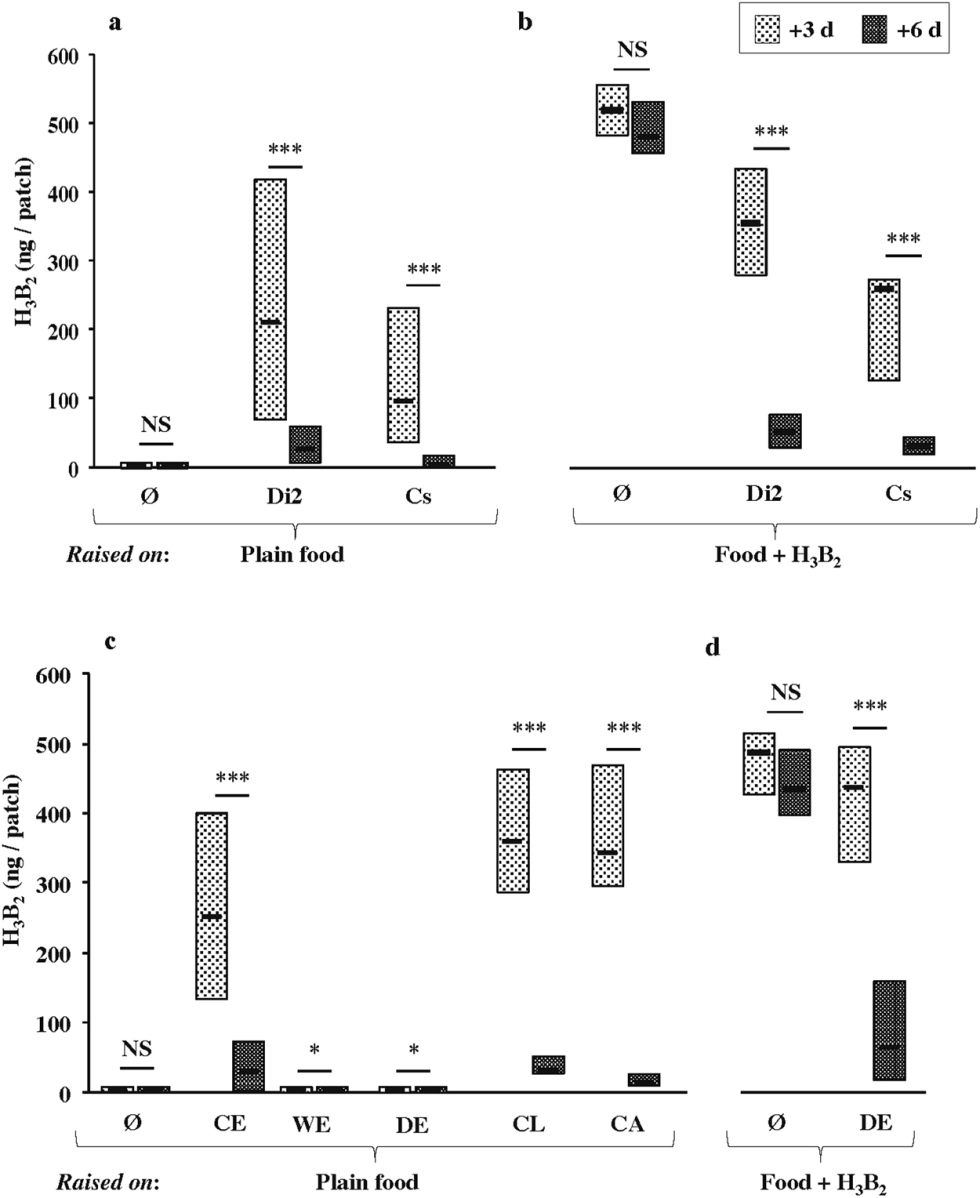
Acetoin content in food patches processed by *D. melanogaster* larvae. We measured the quantity of acetoin in food patches (ng/patch) impregnated by larvae of different strains or conditions. Similar patches were used in parallel for behavioral tests (Fig. 3). Food either contained no larvae (Ø; **a–d**), or was processed either by Dijon 2000 larvae (Di2; **a–d**) or Canton-S larvae (Cs; **a**,**b**). In all tests, we analyzed the acetoin levels in vials 3 days and 6 days after egg-laying (+3d, +6d; light grey and dark grey shaded bars, respectively). We tested the effect of control larvae either on plain food (**a**) or on food mixed with 0.02% acetoin (Food+H_3_B_2_; **b**). We also tested the effect of Di2 larvae resulting of control eggs (CE), or of washed eggs (WE), or of dechorionated eggs (DE) either in plain food (**c**) or in “Food+H_3_B_2_” (**d**). We also tested the effect of an extract of crushed larvae (CL) or crushed adults added in plain food (**c**). Data are shown as box plots representing the 50% median data (second and third quartile separated with a small horizontal bar indicating the median value). For each graph, the quantitative variation of each compound was tested using a Mann-Whitney test (***p < 0.001, *p < 0.05; NS: not significant; N = 10–77).

**Figure 2:**
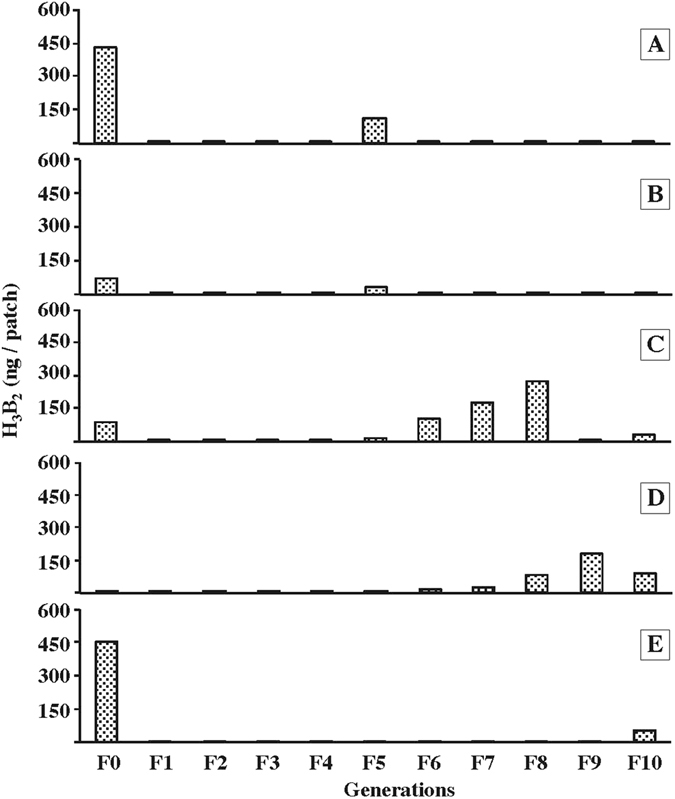
Variation of acetoin content during 10 generations. We simultaneously measured acetoin levels (ng/patch) in food patches processed by larvae in five Di2-derived lines (A–E; +3d vials) selected from a larger sample (F0; Fig. S2). In each line, acetoin production was measured during 10 successive generations (F1–F10). At each generation, progenitors flies inducing the next generation were kept only two days for egg-laying on the food in order to reduce microorganism transmission (see Material and methods).

**Figure 3:**
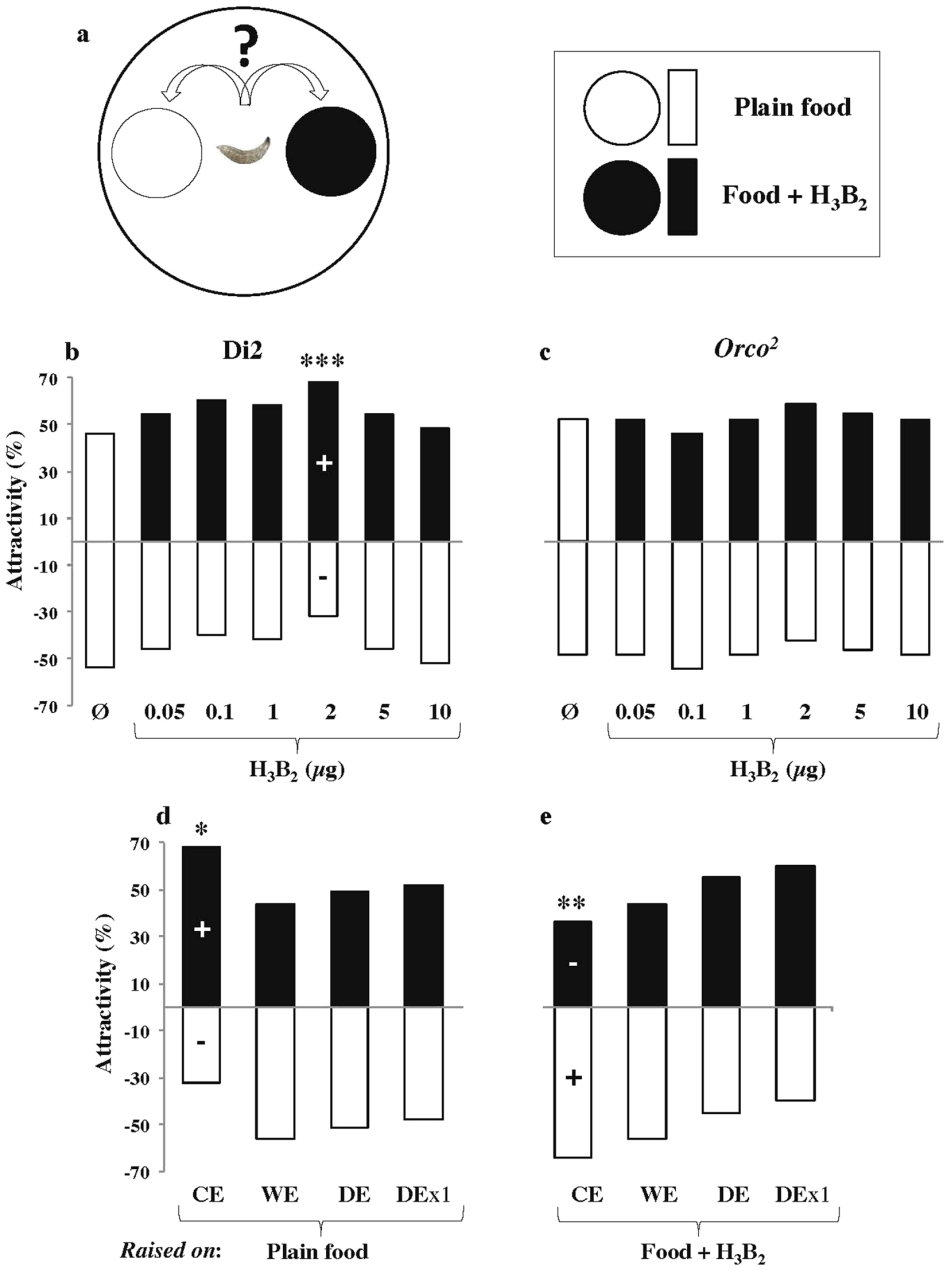
Acetoin preference in Di2 and mutant larvae. (**a**) Each individual early third instar larva was given a binary choice between two food patches either impregnated with plain food (empty circle and bars) or with acetoin-rich food (Food+H_3_B_2_; filled circle and bars). We tested Di2 larvae (**b**,**d**,**e**) and anosmic *Orco* ^2^ mutant larvae (**c**). Each double-sided bar represents the frequency for the first chosen food in the binary choice test. Acetoin was either added at variable quantities: 0.05–10 µg (**b**,**c**) or only 2 µg dose (**d**,**e**). We also performed the control experiment associating two patches of plain food (Ø; **b**,**c**). Larvae were either raised on plain food (**b–d**) or on acetoin-rich food (**e**). We tested larvae resulting of control eggs **(**all tests in **b**, **c**; CE in **d**,**e**) and of manipulated eggs (washed: WE; dechorionated: DE; dechorionated + raised in isolation DEx1; **d**,**e**). For each test, significant differences for binary choice between the two food patches were detected with a Fisher exact test (***p < 0.001; **p < 0.01; *p < 0.05; N = 50 except for **b**-2 µg, **d**-DE, **e**-CE, **e**-DE experiments: N = 100). The “**+**” and “**−**” signs within bars indicate significantly increased and decreased preference for acetoin when all responses corresponding to a complete set of experiment were simultaneously tested (chi-square and post-hoc cell partitioning tests; *p* < 0.05).

**Figure 4:**
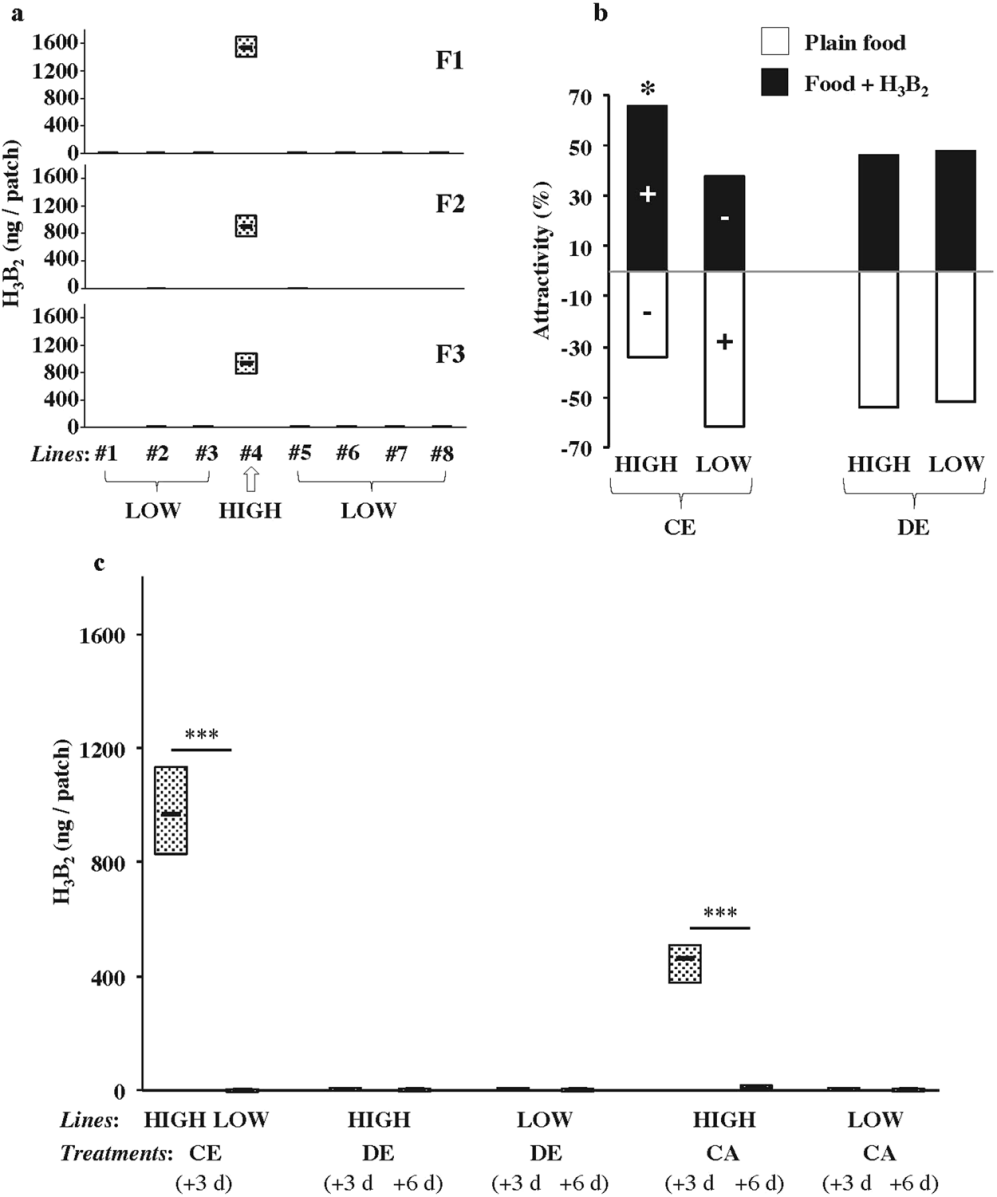
Acetoin production and preference in freshly caught lines larvae. Eight lines sampled in 2016 (Dijon 2016 = Di16) were characterized during their first three generations in the lab (F1–F3). (**a**) The measure of acetoin content (ng/patch) revealed that 7 lines had very low levels (#1–3, #5–8: LOW) whereas one line had a high acetoin level (#4: HIGH). These differences remained very stable, at least until F3. (**b**) F3 larvae of the HIGH and LOW lines were tested for their food preference. Larvae resulting of control eggs (CE) diverged for their response differently to larvae resulting of dechorionated eggs (DE). The “**+**” and “**−**” signs within bars indicate significantly increased and decreased preference for acetoin, when all responses corresponding to a complete set of experiment were simultaneously tested (chi-square and post-hoc cell partitioning tests; *p* < 0.05; N = 50). A significant difference for binary choice between the two food patches was also detected with a Fisher exact test (*p < 0.05). (**c**) Differences for acetoin production. Only HIGH line individual larvae (resulting of control eggs: CE) and adults (crushed and mixed in plain food: CA) produced high acetoin levels in +3d vials. In both cases, the levels in +6d vials strongly decreased. In all other tests (performed with other conditions and vial age) showed no, or very low, acetoin production. Significant differences were detected with a Mann-Whitney test (***p < 0.001; N = 5–10). Note also that all LOW lines were pooled (**b**,**c**).

During the past years (2013–2016), we often noted quantitative variation for the level of acetoin in the food processed by several *D. melanogaster* wild-type lines. For example, the Dijon2000 line (Di2) showed relatively high acetoin levels in 2013 and 2014 (during which all experiments shown on Figs 1–3 were performed), and a drastic decrease in 2015 (Fig. S1). More recently, we sampled freshly-caught lines (Dijon2016 = Di2016; Fig. 4) which also showed a marked inter-line variation.

### Effect of larval development on acetoin production

We first measured whether the acetoin levels could also vary during development with larvae either raised on plain food (Fig. 1a) or on food mixed with 0.02% synthetic acetoin (“food+H_3_B_2_”; Fig. 1b). Plain food vials without larvae (Ø) kept 3 days (+3d; N = 24) and 6 days (+6d; N = 15) showed very low acetoin levels [median values (mv): 1.1 and 1.0 ng, respectively] and no age-related variation (*p* = NS; Fig. 1a). However, plain food vials with Di2 or Canton-S (Cs) larvae contained substantial amounts of acetoin in +3d vials which strongly decreased in +6d vials: in Di2 [mv: 205.0 ng (N = 77) and 26.8 ng (N = 49) respectively; *p* < 0.001] and in Cs [mv: 98.2 (N = 20) and 5.5 ng (N = 20) respectively; *p* < 0.001]. Note that +3d vials mostly contained first and second instar larvae (L1–L2) whereas +6d vials mostly contained third instar larvae (L3).

The control experiment (Ø) performed with “food+H_3_B_2_” revealed no acetoin change between +3d and +6d vials (mv: 501.2 and 461.8 ng, respectively; *p* = NS; N = 11, Fig. 1b). However, the introduction of Di2 or Cs larvae in “food+H_3_B_2_” vials induced a strong acetoin decrease between +3d and +6d vials (mv for Di2: 353.0 to 48.5 ng; N = 12, for Cs: 255.6 to 30.0 ng; N = 7, respectively; *p* < 0.001 for both strains). The comparison between the two food types (for each respective strain and age) revealed no difference except in Di2 +3d “plain food” and Cs +6d “food+H_3_B_2_” vials where acetoin levels dropped compared to Di2 +3d “food+H3B2” (*p* < 0.05) and Cs +6d “plain food” vials (*p* < 0.01), respectively.

### Microorganisms involved in the dynamic change of acetoin

To see whether the biphasic variation of acetoin production during larval development was the result of the activity of microorganisms involved in its synthesis and/or in its degradation, we manipulated the eggs or their environment. First, to test the role of the egg chorion envelope, we either washed Di2 eggs (WE; N = 16; Fig. 1c) or removed their chorion (dechorionated eggs: DE; N = 20). Both WE and DE were subsequently placed onto plain food. Compared to control eggs (CE: 248.1 ng at +3d, N = 37; 26.3 ng at +6d, N = 18), the food processed by WE- and DE-derived larvae contained minute acetoin levels at +3d (mv: 2.8 and 1.2 ng, respectively). A slight decrease was noted in WE and DE +6d vials (mv: 0.8 for both tests; *p* < 0.05) while Ø vials showed equally low acetoin levels at +3d (1.0 ng, N = 28) and at +6d (0.9 ng; N = 13).

To further test the activity of acetoin-related microorganisms associated with Di2 larvae or adults, we either crushed whole L1–L3 larvae (CL; N = 24), or adults (CA; N = 20; Fig. 1c), and added the resulting extract in plain food. In CL and CA experiments, the high acetoin level detected in +3d vials (mv: 367.5 and 355.2 ng, respectively) strongly decreased in +6d vials (mv: 36.5 and 19.9 ng, respectively; *p* < 0.001). Therefore, microorganisms carried by larvae and adults are able, by themselves, to induce the biphasic variation of acetoin similarly to microorganisms hosted by intact control larvae. Moreover, on “food+H_3_B_2_” DE-derived larvae induced a strong acetoin decrease between +3d and +6d (mv: 434.4 to 62.8 ng; N = 25; *p* < 0.001) differently to the control test (Ø) where no acetoin decrease was noted between +3d and +6d (mv: 477.5 to 422.6 ng; N = 10; Fig. 1d).

### Inter-population and –generation variation of acetoin

Next, to sample acetoin variation within a single lab-established strain, we split the Di2 strain into 18 similar-size lines. These lines were simultaneously reared on plain food and their acetoin profiles were measured in +3d and +6d vials (A to R; Fig. S2). Moreover, to investigate for a potential parental age effect, we compared the acetoin profile in larval progeny left by the same female progenitors when 1–2 days old (top histograms) and 3–4 days old (bottom histograms). While no parental-age effect was detected either in +3 d (W = 120; *p* = 0.139) or +6 d vials (W = 89; *p* = 0.896), a general “+3 d to +6 d” decrease was noted both for younger and older progenitor females (W = 151; *p* = 0.005 and W = 170; *p* = 0.0002, respectively).

We also tested the inter-generational variability of acetoin production in five lines retained for their diverse acetoin profiles (A–E; Figs 2 and S2). To obtain and maintain acetoin-free lines, progenitor flies were transferred during 10 generations (F1 to F10) onto fresh food every two days (for the sake of clarity, we only measured +3d vials induced by younger progenitor females). This procedure was effective in drastically decreasing and maintaining acetoin production at a low level in the five lines during the F1–F4 generations, but after the F5 generation, acetoin level started to show variations, both increases and decreases, without line synchrony.

### Larval behavioral response to acetoin: effect of early exposure and olfaction

The second main aspect of our study consisted to determine the behavioral effect induced by the exposure to the acetoin produced by larvae. More precisely, we measured food preference in individual larva presented with the binary choice test “plain food *vs*. food+H_3_B_2_” (Fig. 3a). Given that acetoin is a volatile molecule, we measured two parameters representative of larval olfactory response: (*i*) the first food chosen and *(ii)* the time taken to reach it. Di2 larvae raised in control conditions (CE) and tested with different acetoin doses only showed a significant preference for 2 µg acetoin (N = 100; *p* = 0.0009; Figs 3b and S3a). In contrast, olfactory-defective *Orco* ^2^ mutant larvae—raised and tested in similar conditions—showed no preference (*p* = ns; Figs 3c and S3b).

In the two other experiments, we compared the response to “food+2 µg H_3_B_2_” (the preferred dose) of Di2 larvae originating from manipulated eggs and either raised on plain food (Figs 3d and S3c) or on “food+H_3_B_2_” (Figs 3e and S3d). Larvae derived from control eggs (CE) showed a very different response according to their exposure: larvae raised on plain food were attracted (N = 50; *p* = 0.015)—whereas those raised on “food+H_3_B_2_” were repulsed (N = 100; *p* = 0.006)—by acetoin-rich food. Differently, on both food types, larvae either derived from washed (WE), dechorionated (DE), or dechorionated/isolated eggs (DEx1) showed no preference (*p* = ns). In all behavioral tests, no global difference was noted between the time taken by larvae to reach either type of food, and this was likely due to the high variability of responses (Figs S4 and S5).

### Natural variation of larval acetoin production and preference

Since the phenotypes shown by lab-established lines may result of lab adaptation, we tested acetoin production and preference in larvae from freshly-caught lines (Di2016). Their analysis, performed at the F1, F2 and F3 generations, reveals that most (7/8) of these Di2016 lines produced minute quantities or undetectable levels of acetoin (mv = 1.8ng; N = 8; LOW) whereas one line showed high acetoin levels (#4; mv: 953.5 ng; N = 8; HIGH; Fig. 4a). At F3 generation, the behavioral response of CE-derived larvae, tested in the binary food-preference assay, revealed a strong difference between the two types of lines: HIGH larvae were attracted—whereas LOW lines (pooled) were repulsed—by acetoin-rich food (N = 50; *p* = 0.033; Figs 4b and S3e). Differently, larvae resulting of dechorionated eggs (DE) showed no preference in either HIGH or LOW lines (*p* = ns). Both lines produced very low acetoin levels in +3d and +6d vials (Fig. 4c). Also, a crushed adult extract (CA; N = 10) mixed with plain food induced a biphasic variation in the HIGH line between +3d and +6d vials (mv: 450.1 and 12.2 ng, respectively; N = 10; *p* < 0.001; Fig. 4c) but not of the LOW lines.

## Discussion

In nature and in the lab, the quantity of acetoin left in the food by larvae can largely vary between populations, generations and during larval development. When exploring the origin of this variation, we found a relationship between the level of acetoin naturally produced by larvae and their olfactory preference for acetoin. This indicates the existence of an experience-dependent conditioning.

Lines derived from a single lab-established stock (Di2) showed unpredictable acetoin variations (*i*) at a given generation and (*ii*) between generations. This effect maybe explained by the substantial variability of gut microbiota between natural and laboratory Drosophila populations^13, 26, 27^. The microbiota composition shows more similarity between natural populations and species feeding on the same sources while it varies more between lab-reared lines^13, 17^. This indicates that the conditions of laboratory maintenance can affect the microbiota composition of Drosophila lines^14, 28^, especially in the lines kept on axenic food, or on food containing a broad-spectrum antimicrobial. Our Di2 lines either raised on food with/or without methylparaben (antifungal) showed no quantitative difference for acetoin production (data not shown). When flies are kept longer in the same food vial, they may contain more bacteria due to repeated “feces excretion/reabsorption” cycles^7, 29, 30^, thus likely impacting the acetoin level. If frequent adult transfer to fresh food vials allowed us to rapidly decrease acetoin levels, this effect was not permanent (Fig. 2).

Lines producing substantial levels of acetoin showed an early larval increase phase (L1–L2) followed by a decreased phase (L3). Acetoin increase could be related to the enhanced activity and multiplication of microorganisms during the L1–L2 phase whereas acetoin decrease could be due to (*i*) decreased available resources or to a change (*ii*) of internal physiology, such as *pH* ^31^ or (*iii*) of microbiota composition during L3 development. We experimentally separated acetoin-producing and degrading microorganisms associated with eggs. Microbes involved in the increased production phase are spread over the chorion^7^ explaining why washed or dechorionated eggs (WE, DE) produced no—or minute amounts of—acetoin (Figs 1c and 4c). The decreased acetoin level induced by DE-derived larvae in acetoin-rich food indicates that microbes involved in the degrading phase are not associated with the egg chorion (Fig. 1d). Our data also indicate that larvae and adults carry the microorganisms involved in both phases (CL, CA; Figs 1c and 4c). Given that the yeast used in our plain food is inactivated, the initial synthesis of acetoin may be the result of the activity of the two *D. melanogaster* prominent bacteria species, *Lactobacillus plantarum* and *Acetobacter pomorum* ^14, 31–33^. On the other hand, acetoin catabolism may be governed by several enzymatic systems leading to the production of various derived chemicals into the food often including 2,3-butanediol^34, 35^. However, we only detected minute amounts of this compound (<2 ng/patch) and found no co-variation with acetoin (data not shown).

Gut-associated microorganisms can elicit biosynthesis or catabolism processes yielding pheromonally-active compounds^36^. Acetoin—alone or mixed with other compounds—can attract various adult insects including Scarabaeidae, Dictyopterae, Coleopterae, Psocopterae^36–40^, and also *D. melanogaster* and *D. suzukii* adults^1–3, 23^. Acetoin is a ligand for various olfactory sensory neurons^41–45^. In *D. melanogaster* adults, acetoin perception is processed by the VA2 antennal lobe glomerulus also involved in the close-range attraction of flies to vinegar^46^. Among olfactory receptor proteins (Ors) expressed in both adult and larva, Or2a and Or7a can respond to acetoin^47, 48^. This explains why *Orco* ^2^ mutant larvae showed no preference to acetoin-rich food (Fig. 3c). Differently, *Drosophila simulans* and *D. buzzatii* larvae showed no preference to acetoin-rich food^49^. Our findings fits well with the attraction of *D. melanogaster* larvae to uncharacterized volatile(s) produced by laboratory food mixed with *L. plantarum* bacteria^6^.

Our data reveals the existence of a relationship between the level of acetoin produced by larvae (and to which they are exposed) and their behavioral response to this compound. Acetoin preference was only shown by wild-type larvae producing substantial amounts of acetoin and raised in control conditions (e.g. mass-reared larvae kept in plain food and resulting of intact eggs; Figs 3b and 4b). In contrast, the manipulation of eggs (WE, DE, DEx1; Figs 3d and 4b) or of their environment (addition of synthetic acetoin; Fig. 3e) strongly impaired the larval response. More precisely, larvae derived of the various egg treatments produced no, or very little acetoin and were indifferent to acetoin (Figs 1c and 4c). Very differently, larvae exposed to a homogeneously high level of acetoin showed a repulsive behavior (Fig. 3e). This indicates that the early developmental exposure to acetoin subsequently affects larval preference to this compound. This effect was confirmed with freshly caught lines diverging for their acetoin production (HIGH *vs* LOW; Fig. 4). It is not clear yet whether such experience-dependent conditioning results of an early imprinting effect or of some associative learning process^50^. This conditioning effect can vary between Drosophila species: early developmental exposure to larval-processed food strongly conditioned *D. simulans* and *D. buzzatii* larvae to prefer their own species-labelled food^51^ while it induced a strong aversion in wild-type *D. melanogaster* larvae^52^. This repulsive response was abolished with specific antibiotic treatment^52^. This suggests that the aversive effect induced by the complete mixture of volatile compounds produced by *D. melanogaster* larvae masks the attractive effect elicited by naturally-produced acetoin. This also explains why we did not use antibiotic treatment to cure our lines given the undesired effects induced on larval preference^52^.

The variability of our freshly caught lines suggests that the natural food sources, on which larvae develop, contain variable acetoin levels^7, 30^ (Fig. 4a). We hypothesize that the odors resulting of the activity of yeast and/or bacteria colonies left on food sources by the feces of pioneer insects^3, 19, 21, 53^ elicit fly aggregation and egg-laying^54, 55^. On these sources, developing larvae produce acetoin and other volatile compounds that will in turn attract more larvae^56^ (this study) and adults^2, 12, 57^. Once the colonized source of food contains a “too high” level of acetoin, negatively conditioned larvae search for alternative acetoin-free or -low spots to develop and possibly reproduce, thus initiating a new colonization cycle. We do not know yet whether early developmental exposure to acetoin can change Drosophila adult preference to acetoin-labelled food sites similarly to the effect described with dietary fatty-acids^58^.

Overall, the presence and activity of microbiota at most insect developmental stages is not only critical for the production of volatile metabolites conditioning food choice, but also for many other critical fitness-related traits including growth, immunity, nutrition, kin recognition and mate preference^8, 14, 59–63^.Our study completes this picture and suggests that the natural intraspecific variation of microbiota in insect species can affect their ecological structuration and, on a longer period of time, their evolution.

## Materials and Methods

### Drosophila culture

*D. melanogaster* strains were raised in 150 ml glass vials containing 50 ml of inactivated yeast/cornmeal/agar medium and kept in a breeding room at 24.5 ± 0.5 °C with 65 ± 5% humidity on a 12:12 h light/dark cycle (subjective day from 8:00 am to 8:00 pm). Unless indicated, flies were transferred every two days to avoid larval competition and to regularly provide abundant progeny for testing. All behavioral experiments were performed under similar conditions. We used Dijon 2000 (Di2), a wild-type strain maintained in our lab for 15 years, which showed very stable behavioral performances^52^, the old-established Canton-S strain (Cs), and several freshly caught lines (Dijon 2016: Di16). We also tested the *Orco* ^2^ mutant line (Bloomington stock #23130) in which larvae and flies deprived of the *Or83b* olfactory co-receptor show defective chemosensitive behavior^64^. To obtain a genetic background similar to that of the Di2 strain, the *Orco* ^2^ mutation was introgressed into the genome of the Di2; w^1118^ strain during five repeated backcross generations. For each of the 18 lines established from the parental Di2 strain, 50 young females and 100 young males (<12 h) were kept 48 h to allow mating and egg-laying in a vial containing 4 g of plain-food. After 2 days, adults were transferred in a new vial to allow a second round of egg-laying during 48 h. Then, the level of acetoin was quantified in these vials after 3 and 6 days of larval development (with a single measure for each vial).

### Chemical analysis

We analyzed the chemical composition of the food either plain or processed by Drosophila larvae, 3 and 6 days after egg-laying (+3d, +6d; vials mostly contained L1+L2 or L3 instar larvae, respectively). The food patches to be analyzed and tested were always impregnated following a procedure described earlier^49^. Acetoin was identified by GC-MS, using its retention time and its fragmentation pattern; diagnostic ions were compared with both the NIST/EPA/NIH library and the mass-spectrum of the synthetic chemical standard (Sigma-Aldrich, St Quentin Fallavier, France) analyzed under the same conditions. For quantitative analyses, the response factors of n-pentadecane and acetoin (H_3_B_2_) were determined at 1, 5, 10, 25, 50, 100, 200, 500 and 1000 ng. Each set of measures was performed several times separated in time by few weeks to few months.

### Egg, larval and adult treatments

*Control eggs (CE)*: 50 young females and 100 young males (<12 h day-old) were kept during two days to allow egg-laying in a vial containing 4 g of control plain food (Ø) or 0.02% H_3_B_2_ supplemented food (e.g., 200 ng acetoin/mg plain food). After removing the adults, the concentration of acetoin was estimated in vials after 3 and 6 days of incubation at ±25 °C.

*Washed eggs (WE)*: eggs (<6 h) were rinsed five times in fresh sterile deionized water. Then, about 200 eggs were transferred in a vial containing 4 g of plain-food mixed, or not, with 0.02% H_3_B_2_. Here, the amount of acetoin was also quantified in +3d and +6d vials.

*Dechorionated eggs (DE)*: eggs (<6 h) were rinsed five times in fresh deionized and sterile water. Then eggs were dechorionated by immersion for a few minutes in a 3% solution of sodium hypochlorite, followed by three washes with sterile deionized water. About 200 eggs were transferred in different vials as described above and acetoin was quantified in +3d and +6d vials. We also tested the response of DE-derived larvae raised in isolation (DE*x1*). In this case, freshly laid eggs (<6 h) were dechorionated and individually deposited in a fresh food vial (either containing Plain food or Food+H_3_B_2_) and kept until early L3 stage for testing.

*Body extracts*: whole bodies of fresh anesthetized animals (5 min at −20 °C) corresponding to 3 g of washed larvae (L1–L3) or 4 g of mixed adults (2–4 day-old) were crushed using a sterile grinding glass apparatus in 30 or 40 ml de-ionized sterile water, respectively. Resulting extracts were filtrated through glass-wool to retain intact tissues and 1 ml of each solution was mixed (using a spatula) with 4 g of plain food in a vial. These vials were maintained at 24.5 ± 0.5 °C until being used.

### Behavioral tests

We always used early 3^rd^ instar larvae taken from mass-reared cultures (unless otherwise specified). We followed a similar procedure as that previously described^49^. Before the tests, patches were dipped during 2 hours in plain food and then impregnated either with 5 µl hexane alone or mixed with 0.05–10 µg H_3_B_2_. Patches were always used after solvent evaporation (15 sec at room temperature). Our binary choice test generally involved one “plain food” patch paired with a “food+H_3_B_2_” patch that were alternatively placed in diametrically opposite zones separated by a distance of 30 mm. Each larva was transferred, using a fine brush, halfway between the two patches and a lid covered the dish to avoid excessive evaporation of the tested substances. For each larva, we noted the first food patch chosen and the time necessary to reach this patch. All observations were performed during 30 min under white light at 24.5 ± 0.5 °C. Control experiments consisted in a pair of plain food patches. Control and experimental tests were simultaneously performed. Fifty to 100 replicates were performed depending on the experiment.

### Statistics

Acetoin level measurements were carried out in vials containing a similar number of larvae according to both larval stage and maternal age.

For each studied group, we compared the distribution of H_3_B_2_ amounts between +3d and +6d vials using a Mann-Whitney test (*p* < 0.05). For each behavioral test, we assessed the statistical difference for larval distribution between the two food patches using a Fisher exact test (*p* < 0.05). For each complete set of behavioral tests, a post hoc cell partitioning analysis and a chi-square test (*p* < 0.05) were used to compare the distribution between the different groups of larvae simultaneously tested (Figs 3b–e and 4b).

## Electronic supplementary material

Supplementary information
