# Supplementary material for: Maternally-transmitted microbiota affects odor emission and preference in Drosophila larva
Source: Sci Rep. 2017 Jul 20;7:6062. doi: 10.1038/s41598-017-04922-z (PMC5519639; doi:10.1038/s41598-017-04922-z)
Supplement: Supplementary file 1 — Supplementary information [file 41598_2017_4922_MOESM1_ESM.pdf]

SUPPLEMENTAL INFORMATION FOR:

Maternally-transmitted microbiota affects odor emission and preference in Drosophila larva

Jean-Pierre Farine, Wafa Habbachi, Jérôme Cortot, Suzy Roche, Jean-François Ferveur

SUPPLEMENTARY FIGURE LEGENDS

Supplementary Figure 1. Median acetoin production in Dijon2000 strain during three successive years. Data are shown as box plots representing the 50% median data (or second and third quartile with the median value shown as a small horizontal bar). N=10-31. Measures were carried out during the years 2012, 2013 and 2014.

Supplementary Figure 2. Acetoin production in 18 subpopulations derived from the Di2 parental strain. Acetoin (ng/patch) produced by larvae resulting of 50 younger female progenitors (1-2 days old, top histograms) and older female progenitors (3-4 days old, bottom histograms) was measured in +3d and +6d vials (light grey and dark grey shaded bars, respectively).

Supplementary Figure 3. Larval preference index to acetoin mixed with food.
The acetoin preference index was calculated based on the difference of larvae found on acetoin filter paper minus larvae on plain food divided by the sum of larvae found on both filter papers. A positive index indicates a preference to acetoin whereas a negative index indicates repulsion against acetoin (and/or a preference to plain food). A-D panels correspond to Fig. 3b-e, respectively and panel E corresponds to Fig. 4b.

Supplementary Figure 4. Time necessary to reach each food patch in larvae of a wild type and a mutant strain. Larvae of the wild-type Di2 strain (left series of histograms) or of the *Orco*^{2} mutant resulted of non-manipulated eggs raised on plain food. Each individual larva was presented to a binary choice between two food patches impregnated either with Plain food (empty bars) or with acetoin-rich food (Food+H3B2; mixed with variable acetoin quantities: 0.05-10µg; filled bars). Data are shown as box plots representing the 50% median

data (or second and third quartile with the median value shown as a small horizontal bar). We found no significant difference (chi-square test: p>0.05; N=50-100). All data correspond to Figs. 3b, c.

Supplementary Figure 5. Time necessary to reach each food patch in larvae of a wild type resulting of manipulated eggs. Tests were performed as described above. We tested the preference of Di2 larvae resulting of control eggs (CE), or of washed eggs (WE), of dechorionated eggs (DE), and of dechorionated eggs raised in isolation (DEx1) both on plain food (left series of histograms) and on food+H_3B_2 (2µg). A significant difference was detected with chi-square test (* : p<0.05; N= 50-100). All data correspond to Figs. 3d, e.

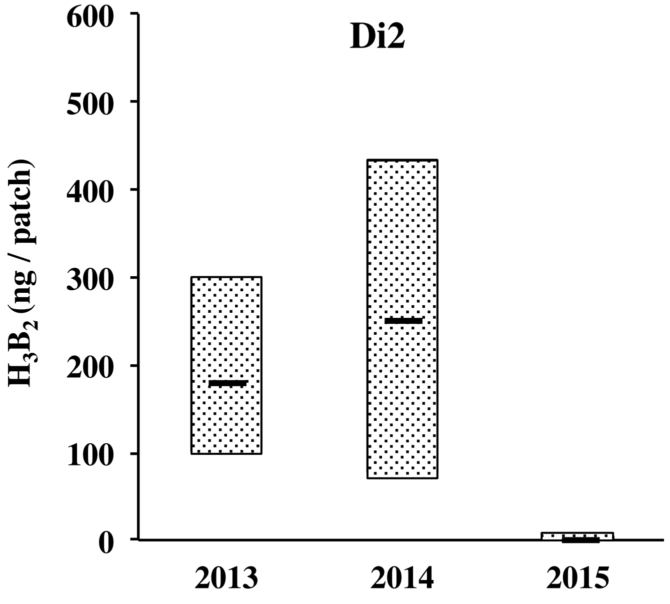

Fig. S1

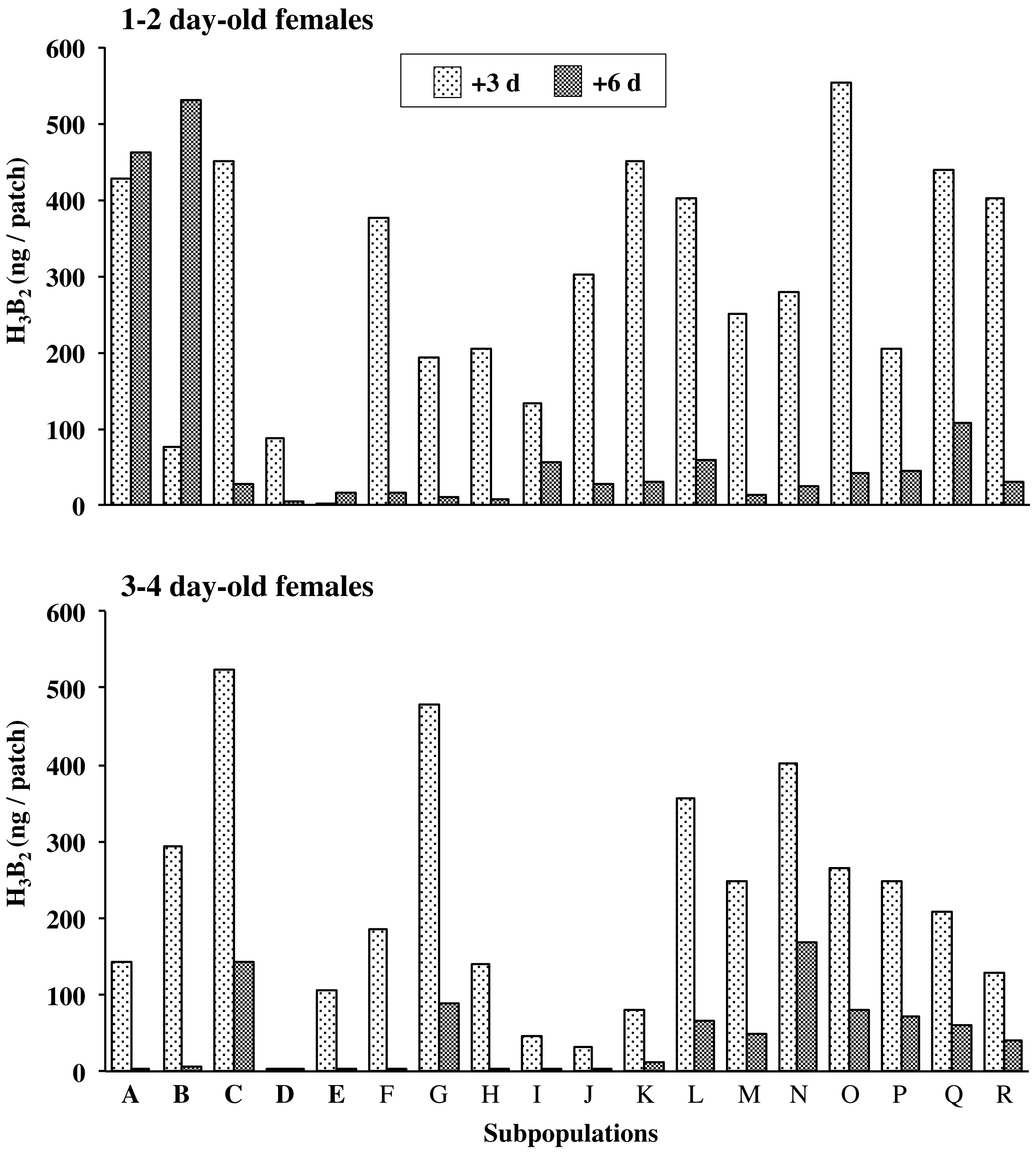

1-2 day-old females
+3 d
+6 d
H3B2 (ng / patch)
0
100
200
300
400
500
600
3-4 day-old females
A B C D E F G H I J K L M N O P Q R
Subpopulations

Fig. S2

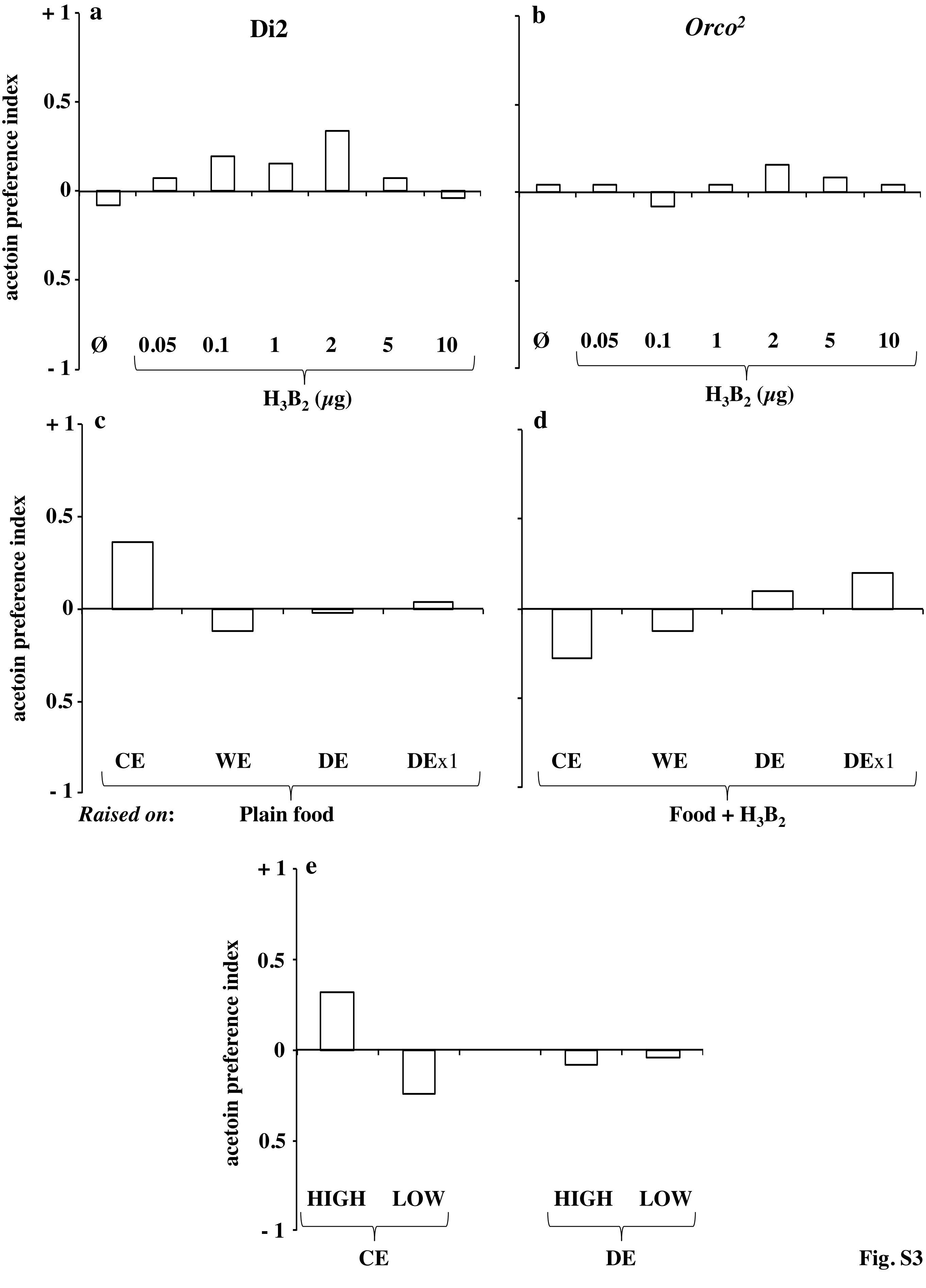
a
Di2
b
Orco2
acetoin preference index
+ 1
0.5
0
0.5
- 1
Ø
0.05
0.1
1
2
5
10
H3B2 (μg)
c
d
CE
WE
DE
DEx1
Raised on:
Plain food
Food + H3B2
e
HIGH
LOW
CE
DE

Fig. S3

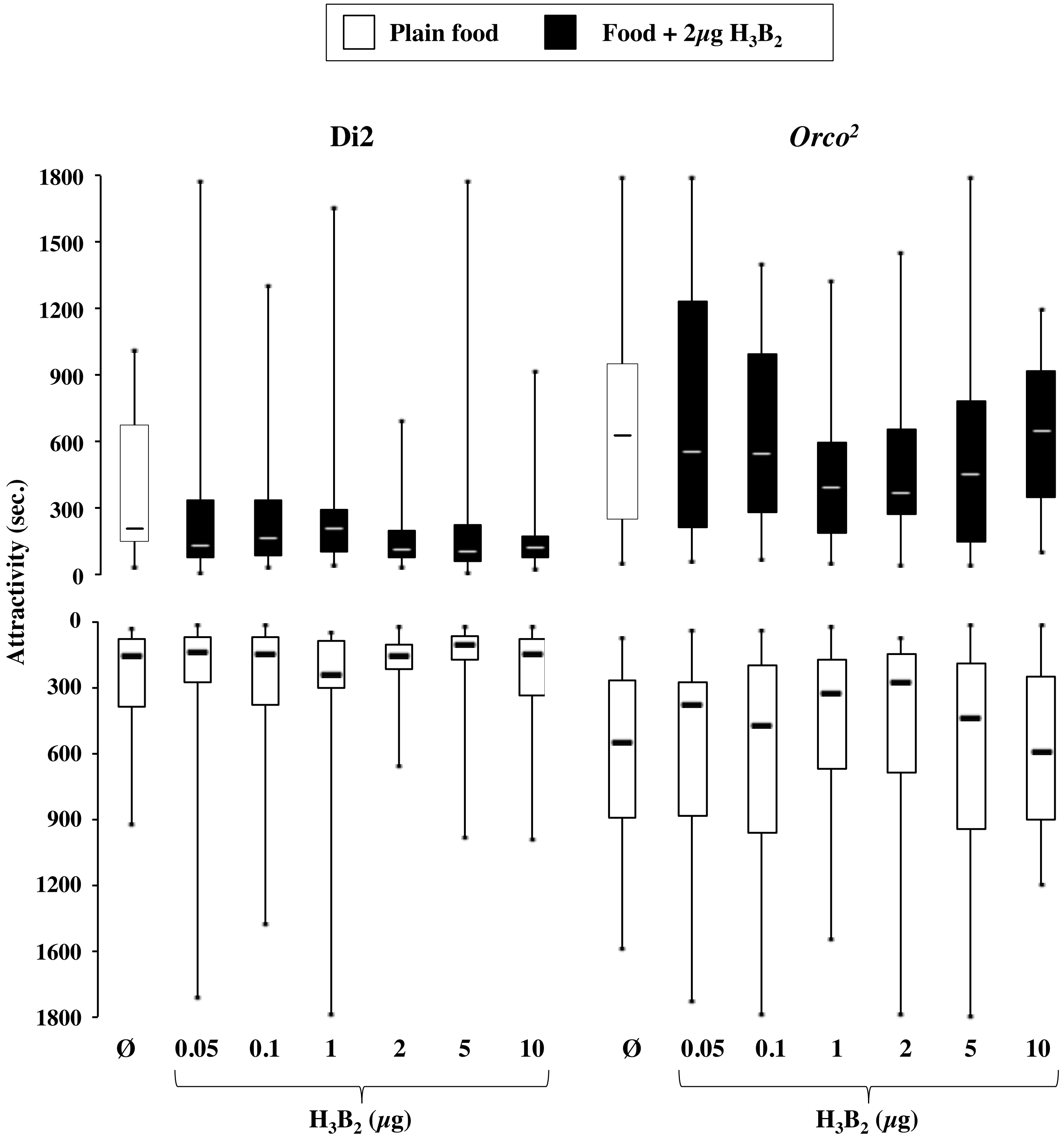

Plain food
Food + 2μg H3B2
Di2
Orco2
Attractivity (sec.)
1800
1500
1200
900
600
300
0
0
300
600
900
1200
1600
1800
Ø
0.05
0.1
1
2
5
10
Ø
0.05
0.1
1
2
5
10
H3B2 (μg)
H3B2 (μg)

Fig. S4

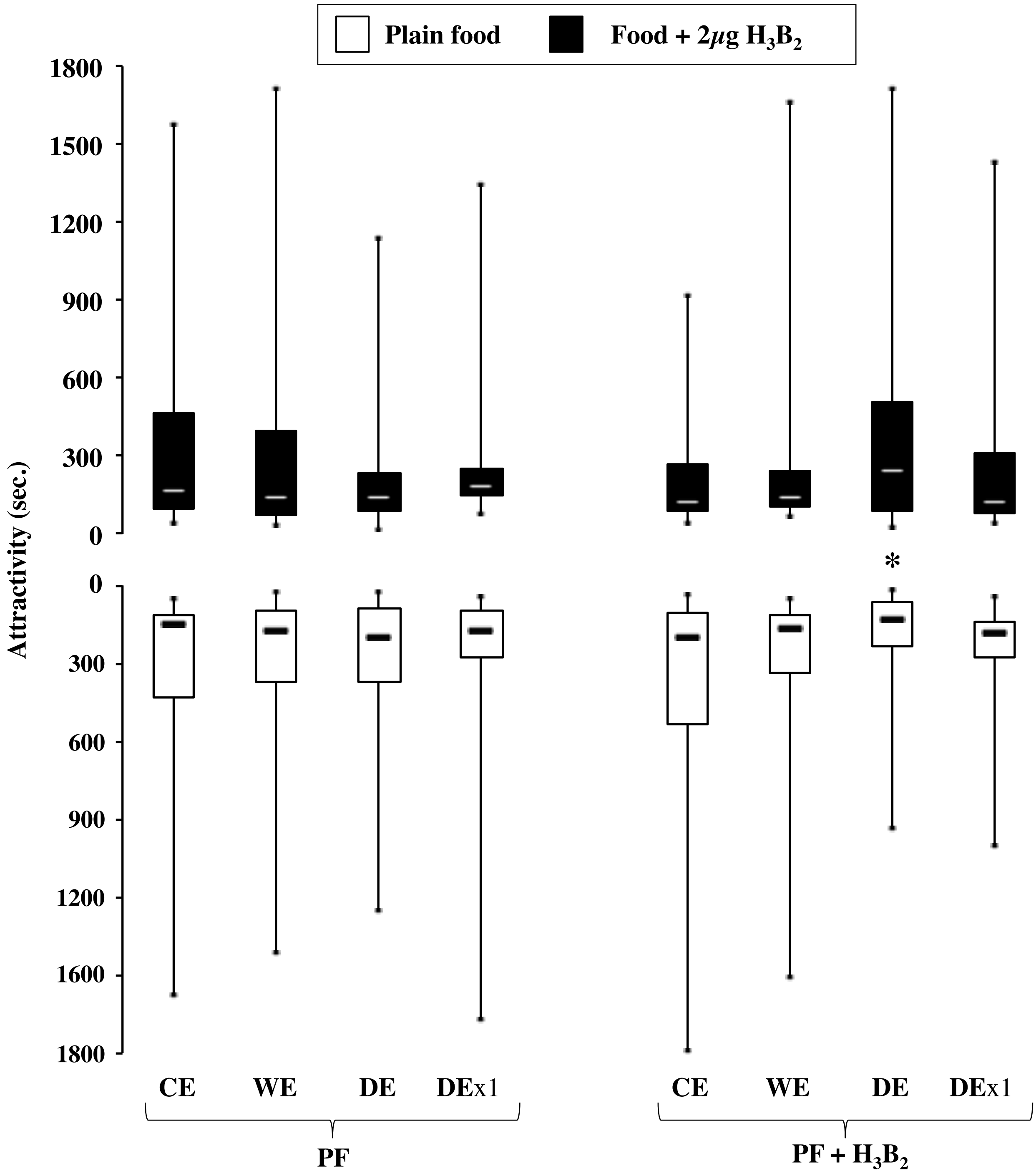
Plain food
Food + 2µg H3B2
Attractivity (sec.)
1800
1500
1200
900
600
300
0
0
300
600
900
1200
1600
1800
*
CE
WE
DE
DEx1
CE
WE
DE
DEx1
PF
PF + H3B2

Fig. S5
